# School bullying victimization-associated anxiety in Chinese children and adolescents: the mediation of resilience

**DOI:** 10.1186/s13034-022-00490-x

**Published:** 2022-06-25

**Authors:** Die Fang, Jin Lu, Yusan Che, Hailiang Ran, Junwei Peng, Lin Chen, Sifan Wang, Xuemeng Liang, Hao Sun, Yuanyuan Xiao

**Affiliations:** 1 Department of Epidemiology and Health Statistics, School of Public Health, Kunming Medical University, 1168 West Chunrong Road, Yuhua Street, Chenggong District, Kunming, 650500 Yunnan China; 2 Department of Psychiatry, The First Affiliated Hospital, Kunming Medical University, Kunming, Yunnan China

**Keywords:** Bullying victimization, Anxiety, Resilience, Mediation, Children and adolescents

## Abstract

**Background:**

School bullying victimization is associated with increased risk of anxiety in children and adolescents. However, little is known about the role of resilience in this association. The purpose of this study was to investigate the possible mediation by resilience in this association in a large group of Chinese children and adolescents.

**Methods:**

A population-based cross-sectional study adopting two-stage simple random cluster sampling was implemented in Yunnan province, southwestern China. A comprehensive questionnaire was used to collect information from the participants. Among all the participants, 4624 were included in the final analysis. Descriptive statistics were used to present general characteristics of the study participants. Univariate and multivariate logistic regression models were adopted to estimate crude and adjusted associations among bullying victimization, anxiety, and resilience. A path model was used to analyze the hypothesized mediation by resilience in the association between bullying victimization and anxiety.

**Results:**

Analytical results of multivariate logistic regression models suggested that bullying victimization was significantly associated with anxiety in Chinese children and adolescents; compared with individuals who had not been bullied, victims of bullying were more likely to experience anxiety symptoms, with an adjusted odds ratio (OR) of 3.02 (95% CI 2.46–3.71). Path analysis revealed a prominent mediating effect of resilience in the association between bullying victimization and anxiety, accounting for 31.89% of the total association. Further analysis indicated that, among the five dimensions of resilience, emotional regulation, interpersonal assistance, and family support were significant mediators, accounting for 30.34%, 10.79%, and 8.35% of the total association.

**Conclusions:**

Our major findings highlighted the promising role of resilience-based intervention measures in reducing the risk of anxiety associated with school bullying victimization in Chinese children and adolescents.

**Supplementary Information:**

The online version contains supplementary material available at 10.1186/s13034-022-00490-x.

## Background

Anxiety is a negative emotion, a subjective experience of tension, apprehension, and worry in the face of uncertainty and various life changes, accompanied by physiological and behavioral changes [1]. The estimated prevalence of anxiety in children and adolescents is high. In the United States, the National Comorbidity Survey (NCS) reported a lifetime prevalence of 31.9% for adolescent anxiety [2]. In China, about 32% of adolescents present anxiety symptoms [3]. Generally, anxiety is elevated during adolescence, mainly due to concerns about relationships; moreover, it can persist over time and affect future life [4]. Adolescent anxiety may have severe consequences: for instance, it is an identified risk factor for self-harm and suicidal behaviors [5].

Bullying refers to repeated and deliberate aggression by using physical or emotional means to control or harm another person, and is a form of damaged peer relationship [6]. The roles involved in school bullying include: being bullied (victims), bullying others (bullies), and bullying others while being bullied (bully-victims) [7]. A European multicenter study reported a bullying victimization rate of 18.2% among children [8]. A meta-analysis showed that the prevalence of bullying victimization among adolescents was 36% [9]. In China, it has been reported that 13.13% of youths were victims of bullying [10]. Previous studies have shown that bullying victimization not only leads to increased behavioral and emotional problems [11], psychiatric symptoms [12], and reduced academic performance [13], but is also associated with increased risk of suicidal ideation and behaviors [14].

Bullying victimization may also increase the risk of anxiety [15]. A British cohort study found that the experience of bullying victimization in childhood contributed significantly to anxiety in the following 2 years [16]. Moreover, anxiety in bullying victims was twice as frequent as in those who had not been bullied [17]. Therefore, it is imperative to reduce the risk of anxiety associated with school bullying victimization in children and adolescents. However, direct intervention on school bullying victimization is likely to be ineffective. First, victims of bullying may choose to hide their experiences for fear of rejection by peers [18]. Second, although there are many proposed intervention measures for bullying in schools, incongruent results have been published, with some studies finding that bullying even increased after intervention [19–22]. Under this scenario, exploring intervenable factors in the association between school bullying victimization and anxiety among children and adolescents will give useful information.

Resilience has been one of the most popular topics in positive psychology in recent years. It refers to an individual’s ability successfully to deal with things or cope with problems [23]. Resilience is a multi-dimensional concept consisting of internal and external factors: internal factors include goal concentration, emotion regulation, and positive cognition; external factors include family support and interpersonal assistance [24]. Among the internal factors, it has been found that emotion regulation is significantly associated with the onset of anxiety [25]. Among external factors, the prominent association between family support and anxiety has also been reported: a previously published meta-analysis revealed that insufficient family support, characterized by less parental warmth, was associated with an increased risk of anxiety in adolescents [26]. Meanwhile, involvement in bullying victimization has been found to be significantly related to a diminished level of resilience in youths [24]. Moreover, one study found that adolescents with higher resilience level experienced less anxiety after school bullying victimization [27], which suggested moderation by resilience in the bullying–anxiety association.

In this study, using a large sample of Chinese children and adolescents, we intended to explore the association between school bullying victimization and anxiety, with a particular focus on the mediating and moderating effects of resilience in this association, a reasonable hypothesis that has not been effectively discussed so far.

## Materials and methods

### Study design

A cross-sectional survey was conducted in Kaiyuan city, Honghe prefecture, Yunnan province, China, from October 19 to November 3, 2020. A two-stage simple random cluster sampling method with probability proportionate to sample size (PPS) design was used: in the first stage, 19 schools (eight primary schools, nine junior high schools, and two senior high schools) were randomly selected from all schools in Kaiyuan; in the second stage, according to the required sample size, 4–6 classes were randomly selected from each of the chosen schools, and all eligible students within the chosen classes were included.

The study protocol was reviewed and approved by the Ethics Review Committee of Kunming Medical University. After obtaining signed informed consent from the legal guardians of the eligible participants, a self-administered questionnaire survey was conducted. Considering that a self-administered survey is prone to missing data, after completion, every questionnaire was carefully checked and reviewed by pre-trained quality control personnel, who were Masters Degree students either in psychiatry or in public health from Kunming Medical University, or local health professionals recruited in Kaiyuan.

### Measurements

The questionnaire we used is a comprehensive instrument collecting the following information: general characteristics (including demographics and details of the family), anxiety, school bullying, resilience, self-harm, mobile phone use, sleep, suicidal ideation and behaviors, life events, etc. In this study, our analysis was based on the former four parts.

### Anxiety

Anxiety was evaluated using the Generalized Anxiety Disorder-7 (GAD-7) scale [28], an instrument which assesses the frequency of anxiety symptoms in the past 2 weeks (not at all, only a few days, more than half of the days, and almost every day), with assigned scores from 0 to 3. The total score for the GAD-7 is 0–21 points, and a cut-off of 4 is usually recommended for screening positive individuals [29]. The Cronbach’s α of GAD-7 for our study sample was 0.907 (bootstrap 95% CI 0.900–0.914).

### Bullying victimization

School bullying was measured using the Chinese version of the Olweus bully/victim questionnaire (OBVQ) [30]. The OBVQ can be divided into three dimensions, each containing two separate questions. All questions are measured by a consistent 5-point Likert style responses (never, once or twice in total, twice or three times a month, once a week, several times a week). Individuals who answered “twice or three times a month” or more frequently for being bullied, bullying others, or bullying others while being bullied were classified as victims, bullies, or bully-victims, respectively [31]. In this study, we only included bullying victims and uninvolved individuals for further analysis; bullies and bully-victims were deleted before the analysis.

### Resilience

Resilience was measured using the Resilience Scale for Chinese Adolescents (RSCA) developed by Hu and Gan [32]. It evaluates the following five dimensions of resilience: goal concentration, emotion regulation, positive cognition, family support, and interpersonal assistance. The RSCA contains a total of 27 questions; each question can be assigned a score from 1 to 5 based on the response, with a higher score indicating a higher level of resilience in general or in a specific dimension. The Cronbach’s α of the RSCA for our study sample was 0.838 (bootstrap 95% CI 0.830–0.845).

### Statistical analysis

Descriptive statistics were calculated to describe the general characteristics of the participants. Crude and adjusted associations among bullying victimization, anxiety, and resilience were analyzed using univariate and multivariate logistic regression models. In addition, the product term of bullying victimization and resilience was included in the multivariate model with anxiety as the dependent variable to evaluate the moderating effect of resilience on the association between bullying victimization and anxiety. Finally, the hypothesized mediating action of resilience in bullying victimization and anxiety was assessed using path analysis. The R software (Version 3.6.2, The R Foundation for Statistical Computing, Vienna, Austria) was used to perform the analysis. Survey data related packages were used to adjust for possible intercorrelation between particpants sampled from the same cluster.

The statistical significance level was set as *p* < 0.05, two-tailed, except for univariate logistic regression, which adopted a comparatively loose criterion of *p* < 0.10. Study participants with missing information in analytical variables were deleted.

## Results

A total of 4883 adolescents were surveyed. After data sorting, 162 students with missing information were excluded. In this study, we only included victims of school bullying, therefore another 97 students were further excluded for being bullies or bully-victims. In the end, 4624 study participants were included in the final analysis, with an effective response rate of 94.70%.

### General characteristics

The major features of the 4624 students included in the final analysis are displayed in Table 1. The distribution of sex was reasonably equal, the average age was 12.98 years, most of the participants were from ethnic minorities (72.5%) and 19.6% were left-behind children. Among the participants, 26.5% (95% CI 20.9%–33.0%) presented symptoms of anxiety, and 12.9% (95% CI 9.9%–17.0%) were victims of school bullying. The median for the total RSCA score was 90; for the five dimensions of resilience, the medians were 18 (goal concentration), 20 (emotion regulation), 14 (positive perception), 21 (family support), and 20 (interpersonal assistance), respectively.

**Table 1:** General features of the study subjects, Kaiyuan, Yunnan, 2020 (*N* = 4,624)

Features	*N* (%)	Mean (SE)/Median (IQR)
Sex		
Boys	2,281 (49.3)	
Girls	2,343 (50.7)	
Age		12.98 (0.41)
Ethnicity		
Han	1,273 (27.5)	
Minorities	3,351 (72.5)	
Grade		
Primary school	1,570 (34.0)	
Junior high school	2,488 (53.8)	
Senior high school	566 (12.2)	
Only child		
No	3,598 (77.8)	
Yes	1,026 (22.2)	
Parents’ marital status		
In marriage	3,922 (84.8)	
Not in marriage	702 (15.2)	
Left-behind children		
No	3,717 (80.4)	
Yes	907 (19.6)	
Anxiety (GAD-7 ≥ 4)		
No	3,400 (73.5)	
Yes	1,224 (26.5)	
Bullying victimization		
No	4,029 (87.1)	
Yes	595 (12.9)	
Resilience		
Combined RSCA score		90 (21)
Goal concentration (Dimension 1)		18 (5)
Emotion regulation (Dimension 2)		20 (8)
Positive perception (Dimension 3)		14 (5)
Family support (Dimension 4)		21 (6)
Interpersonal assistance (Dimension 5) (Dimension 5)		20 (6)
General resilience level		
Low (RSCA ≤ 90)	2414 (52.2)	
High (RSCA > 90)	2210 (47.8)	

### Associations among bullying victimization, anxiety, and resilience

Table 2 shows the crude and adjusted associations among bullying victimization, resilience, and anxiety, with anxiety as the dependent variable. Based on the results of the univariate model, a series of multivariate models were fitted: multivariate models 1 and 2 included bullying victimization and resilience separately; multivariate model 3 included bullying victimization and resilience simultaneously; multivariate model 4 further incorporated the product term of bullying victimization and resilience to estimate possible moderation. The analytical results suggested that bullying victims were more likely to experience anxiety symptoms than adolescents who had not been bullied (adjusted OR = 3.02, 95% CI 2.46–3.71), adolescents with a higher resilience level were less likely to experience anxiety (adjusted OR = 0.27, 95% CI 0.22–0.32), and the moderation by bullying victimization and resilience was not statistically significant. The adjusted associations between bullying victimization and resilience obtained by taking resilience as the dependent variable were estimated also by using logistic regression models, and the results indicated a significant adjusted association (see Additional file 1: Table S1).

**Table 2:** Univariate and multivariable Logistic regression models fitting results for anxiety

Covariates	Univariate model	Multivariate model 1	Multivariate model 2	Multivariate model 3	Multivariate model 4
	Crude OR (90% CI)	Adjusted OR (95% CI)	Adjusted OR (95% CI)	Adjusted OR (95% CI)	Adjusted OR (95% CI)
Sex: girls (Ref: boys)	1.94 (1.68, 2.25)^*^	2.07 (1.70, 2.52)^**^	2.00 (1.61, 2.47)^**^	2.09 (1.67, 2.62)^**^	2.09 (1.67, 2.63)^**^
Age (+ 1 year)	1.29 (1.23, 1.36)^*^	1.13 (1.05, 1.22)^**^	1.14 (1.07, 1.22)^**^	1.14 (1.06, 1.23)^**^	1.14 (1.00, 1.23)
Ethnicity: other minorities (Ref: Han)	1.03 (0.82, 1.28)				
Grade (Ref: primary school)					
Junior high school	2.77 (2.02, 3.81)^*^	2.43 (1.57, 3.76)^**^	1.90 (1.16, 3.11)^**^	2.19 (1.34, 3.60)^**^	2.20 (1.34, 3.62)^**^
Senior high school	4.62 (3.46, 6.18)^*^	3.09 (1.87, 5.12)^**^	2.71 (1.69, 4.34)^**^	3.35 (2.03, 5.34)^**^	3.37 (2.03, 5.59)^**^
Only child: Yes (Ref: No)	1.17 (0.93, 1.47)				
Parents’ marital status: Not in marriage (Ref: in marriage)	1.33 (1.14, 1.55)^*^	1.25 (1.04, 1.51)^**^	1.28 (1.03, 1.60)^**^	1.19 (0.96, 1.48)	1.19 (0.96, 1.48)
Left-behind situation: Yes (Ref: No)	1.17 (0.97, 1.42)				
Bullying victimization: Yes (Ref: No)	2.33 (1.86, 2.91)^*^	3.47 (2.89, 4.16)^**^		3.02 (2.46, 3.71)^**^	2.88 (2.29, 3.62)^**^
Resilience: RSCA > 90(Ref: RSCA < = 90)	0.27 (0.24, 0.31)^*^		0.25 (0.21, 0.30)^**^	0.27 (0.22, 0.32)^**^	0.26 (0.22, 0.32)^**^
Victimization × Resilience					1.19(0.77, 1.86)^**^

^*^p < 0.10

^**^p < 0.05

### Mediation of resilience in school bullying victimization and anxiety

**Fig. 1:**
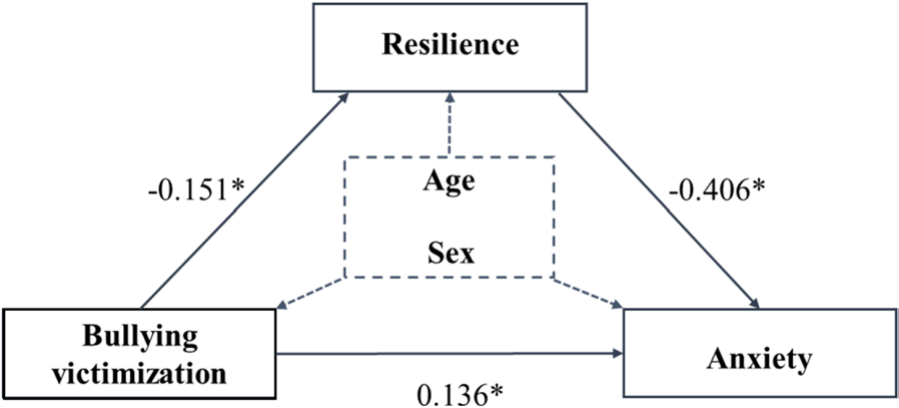
The path model of resilience, bullying victimization, and anxiety. ^*^p < 0.05

The analytical results from multivariate models supported the suspected mediation by resilience in the association between school bullying victimization and anxiety. We constructed a path model to validate this hypothesis (Fig. 1). The analytical results revealed that the indirect association via resilience was 0.061 (calculated as −0.151 × −0.406), it was statistically significant and accounted for nearly one-third (31.89%) of the total association between bullying victimization and anxiety.

**Fig. 2:**
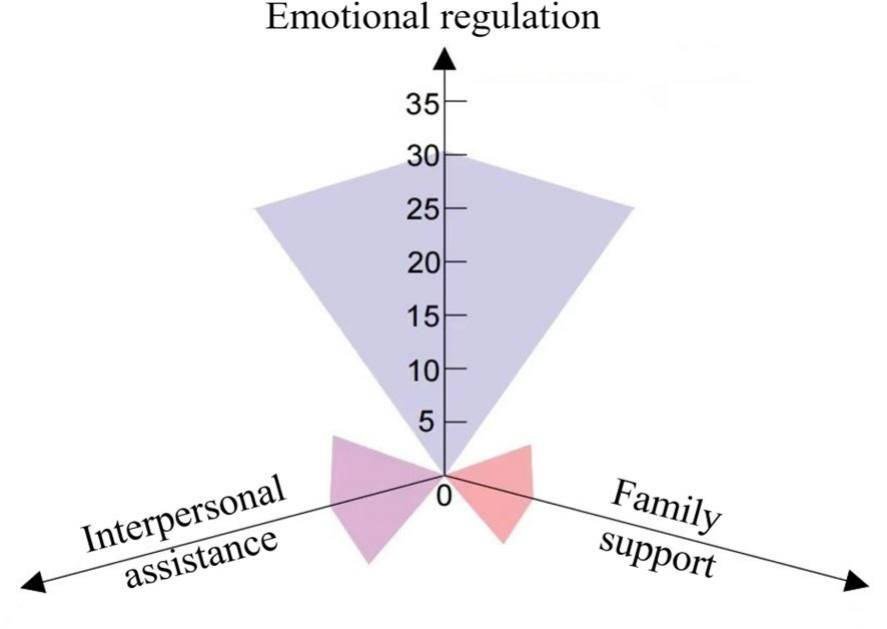
The mediation of different dimensions of resilience in the bullying victimization-anxiety association

We further analyzed the mediation by using the five dimensions of resilience (goal concentration, emotional regulation, positive perception, family support and interpersonal assistance) separately. The analytical results indicated that emotional regulation, interpersonal assistance, and family support were significant mediators, accounting for 30.34%, 10.79%, and 8.35% of the total association between bullying victimization and anxiety (Fig. 2).

## Discussion

In this study, not only did we find a significant association between bullying victimization and anxiety, but we also found that resilience played a prominent mediating role in this important association. Moreover, among the five dimensions of resilience, emotional regulation, family support, and interpersonal assistance presented as significant mediators. These major findings highlighted the significance of resilience in interventions designed to prevent anxiety associated with school bullying victimization in Chinese children and adolescents.

Supportive evidence can be found for this significant mediation by resilience. The anxiety associated with bullying victimization probably can be ascribed to avoidance, feelings of isolation, and lack of friendship [33]. There are three models of resilience that explain how it counteracts the occurrence of negative outcomes: compensatory, protective, and challenge [34, 35]. Bullying is a specific type of interpersonal violence; promoting factors (such as perceived social status) and compensating factors (such as self-efficacy and parental support) can reduce or prevent bullying through compensatory and protective models [36]. It has been documented that a higher level of resilience was associated with a lower level of anxiety symptoms [37]. This was probably because individuals with a higher level of resilience have more promoting and compensatory factors, and therefore may experience fewer symptoms of anxiety even under bullying victimization.

Among all dimensions of resilience, emotion regulation played the strongest mediating role, accounting for nearly one-third of the entire association. Victims of bullying usually report stronger negative emotions and higher levels of emotion dysregulation compared with those who were not bullied [38, 39]. It has been verified that disruption in emotion regulation may increase the risk of internalizing problems in adolescents, leading to depression and anxiety [40]. Longitudinal studies have shown that deficit in emotion regulation predicted anxiety and depression within 5 years, and that systematically improving emotion regulation can prevent the development of anxiety disorders [41, 42]. To strengthen emotional regulation ability, two types of intervention measure can be considered: adaptive emotion regulation strategies (cognitive reappraisal, problem solving, and acceptance) and maladaptive emotion regulation strategies (avoidance, suppression, and rumination). It has been found that both types can contribute to the reduction of depression and anxiety [43]. Moreover, a meta-analysis showed that a combination of cognitive–behavioral interventions had better outcomes than emotion regulation interventions alone [44].

Family support and interpersonal assistance were also significant mediators in the association between bullying victimization and anxiety, although their mediating effect was not as strong as that of emotion regulation. Parental support and interpersonal assistance are vital sources of social support for children and adolescents. A previously published study revealed that they effectively buffered the association between bullying and non-suicidal self-harm, an intimate outcome of anxiety [45]. Longitudinal evidence showed that parental support and interpersonal assistance could reduce the occurrence of negative emotions and the risk of bullying victimization [46, 47]. This section of the results reminded us that promoting family and interpersonal support might also be beneficial in preventing the anxiety associated with school bullying victimization in children and adolescents. For intervention methods, a whole-school strategy which involves parents, peers, and teachers could be considered, because this has already shown a promising effect in reducing school bullying [48–50].

## Limitations

Our study was among the first attempts to discuss the mediating effect of resilience in school bullying victimization and anxiety in Chinese children and adolescents; the large sample size and scientific study design consolidated the validity and reliability of the study results. However, it also had some limitations. First, this was a cross-sectional study, therefore causal inference should be avoided. Second, the self-report method used in collecting data may be prone to information bias. Finally, study participants were chosen from a specific province in southwestern China, and representativeness should be considered when interpreting the results.

## Conclusions

In this population-based cross-sectional study that included a large sample of Chinese children and adolescents, we found that school bullying victimization was significantly associated with anxiety; more importantly, resilience played a role as a prominent mediator in this association, especially for the dimensions of emotional regulation, family support, and interpersonal assistance. Our major findings highlighted the promising role of resilience-based intervention measures in preventing anxiety associated with school bullying victimization in children and adolescents. Future longitudinal studies are needed to corroborate our study results.

## Supplementary Information

**Additional file 1: Table S1.** Univariate and multivariable Logistic regression models fitting results for resilience

## Data Availability

The analytical database of this study can be obtained from the corresponding author under reasonable request.

## Abbreviations

GAD-7: Generalized anxiety disorder-7
OBVQ: Olweus bully/victim questionnaire
RSCA: Resilience scale for Chinese Adolescents
