# Supplementary material for: School bullying victimization-associated anxiety in Chinese children and adolescents: the mediation of resilience
Source: Child Adolesc Psychiatry Ment Health. 2022 Jun 25;16:52. doi: 10.1186/s13034-022-00490-x (PMC9233828; doi:10.1186/s13034-022-00490-x)
Supplement: Supplementary file 1 — Additional file 1: Table S1. Univariate and multivariable Logistic regression models fitting results for resilience [file 13034_2022_490_MOESM1_ESM.docx]

| Table S1 Univariate and multivariable Logistic regression models fitting results for resilience | | |
| --- | --- | --- |
| Features | Univariate model (Event: RSCA≥90) | Multivariate model (Event: RSCA≥90) |
|  | Crude OR (90% CI) | Adjusted OR (95% CI) |
| Sex: Girls (Ref: Boys) | 0.92 (0.79,1.08) |  |
| Age(+1year) | 0.98 (0.91,1.06) |  |
| Ethnicity: Other minorities (Ref: Han) | 0.75 (0.62,0.91) | 0.82 (0.68,0.98) |
| Grade (Ref: Primary school) |  |  |
| Junior high school | 0.71 (0.52,0.98) | 0.69 (0.49,0.99) |
| Senior high school | 1.14 (0.76,1.71) | 1.02 (0.67,1.54) |
| Only child: Yes (Ref: No) | 1.49 (1.26,1.77) | 1.47 (1.23,1.77) |
| Parents’ marital status: Not in marriage (Ref: In marriage) | 0.81 (0.71,0.93) | 0.76 (0.65,0.90) |
| Left-behind children: Yes (Ref: No) | 0.84 (0.70,1.01) |  |
| Bullying victimization: Yes (Ref: No) | 0.51 (0.41,0.64) | 0.49 (0.39,0.62) |
